# Functional Differentiation of BMP7 Genes in Zebrafish: *bmp7a* for Dorsal-Ventral Pattern and *bmp7b* for Melanin Synthesis and Eye Development

**DOI:** 10.3389/fcell.2022.838721

**Published:** 2022-03-15

**Authors:** Xiao-Ru Dong, Shi-Ming Wan, Jia-Jia Zhou, Chun-Hong Nie, Yu-Long Chen, Jing-Han Diao, Ze-Xia Gao

**Affiliations:** ^1^ Key Lab of Freshwater Animal Breeding, College of Fisheries, Ministry of Agriculture and Rural Affairs/Key Lab of Agricultural Animal Genetics, Breeding and Reproduction of Ministry of Education/Engineering Technology Research Center for Fish Breeding and Culture in Hubei Province/Engineering Research Center of Green Development for Conventional Aquatic Biological Industry in the Yangtze River Economic Belt of Ministry of Education, Huazhong Agricultural University, Wuhan, China; ^2^ Hubei Hongshan Laboratory, Wuhan, China; ^3^ CAS Key Laboratory of Tropical Marine Bio-Resources and Ecology, South China Sea Institute of Oceanology, Innovation Academy of South China Sea Ecology and Environmental Engineering, Chinese Academy of Sciences, Guangzhou, China; ^4^ Guangdong Laboratory for Lingnan Modern Agriculture, Guangzhou, China

**Keywords:** *bmp7a*, *bmp7b*, growth difference, eye defect, melanin pigmentation, transcriptome, zebrafish

## Abstract

Bone morphogenetic protein 7 (BMP7) belongs to the transforming growth factor β (TGF-β) family, which not only induces cartilage and bone formation, but also regulates eye development and melanoma tumorigenesis in mammals. In teleosts, BMP7 differentiates into two subtypes, *bmp7a* and *bmp7b*, which have clearly differentiated structures. To fully understand the functional differentiation of *bmp7a* and *bmp7b* in fish species, we successfully constructed *bmp7a* and *bmp7b* gene deletion mutants in zebrafish using CRISPR/Cas9-mediated gene editing technology. Our results showed that *bmp7a* mutation caused abnormal development of the embryo’s dorsal-ventral pattern that led to death; *bmp7b* mutation induced growth inhibition and increased melanin production in the skin and eye of mutants. Histological analysis revealed that melanin in the retina of the eyes in *bmp7b* mutants increased, and behavioral observation showed that the vision and sensitivity to food of the mutants were reduced. Transcriptome analysis of the skin and eye tissues showed that the expression changes of *wnt7ba* and *gna14* in *bmp7b* mutants might promote the increase of melanin. Additionally, the eye transcriptome analysis indicated that changes in the structure of the eyes in *bmp7b* mutants led to defects in phototransduction, and seven DEGs (*rgs9a*, *rgs9b*, *rcvrn2*, *guca1d*, *grk1b*, *opn1mw4,* and *gc2*) were identified as key candidate genes that affected the photonic response of the eyes. The study revealed the functional differentiation of *bmp7a* and *bmp7b* in teleosts and the first report about the inhibitory effect of *bmp7b* on melanogenesis may provide useful information for the future research on human melanoma-related diseases.

## Introduction

Bone morphogenetic protein 7 [BMP7 (OP-1)], a member of the transforming growth factor-β (TGF-β) superfamily, is a 35 kDa homodimeric protein that is involved in the differentiation of mesenchymal cells into osteoblasts and promotes ectopic bone formation (Celeste et al., 1990; Ozkaynak et al., 1990; Ducy and Karsenty, 2000). BMP7 mainly binds to type I and type II BMP receptors expressed on the cell surface to activate receptor-regulated SMADs (R-SMADs, including SMAD1, 2, 3, 5, and 8). The activated R-SMADs interact with SMAD4 (Co-SMAD) to form a complex, which then translocates to the nucleus to regulate the transcription of the target gene (Bragdon et al., 2011). In addition to bone formation, previous studies have proved that BMP7 also has an indispensable role in the growth, differentiation, and apoptosis of various tissues (Ducy and Karsenty, 2000). In humans and mammals, BMP7 is involved in the regulation of hair follicle development and hair growth (Lv et al., 2016), and the size and spatial distribution of hair (Michon et al., 2008). In the early development of birds, BMP7 signaling mediates the ability of the epidermis to respond to inductive signals from the dermis to form feathers and scales (Harris et al., 2004). In recent years, BMP7 was also shown to be involved in regulating the production of melanin and was strongly expressed in metastatic and primary melanoma (Rothhammer et al., 2005; Vitic et al., 2021).

Furthermore, BMP7 is crucial for numerous biological processes in vertebrates. For example, the *bmp7* gene plays an osteoinductive role in the development and reconstruction of human and mouse teeth and alveolar bone (Zouvelou et al., 2009; Kim et al., 2019); 25% of *bmp7* homozygous mutant mice showed small size, eye deformities, kidney hypoplasia, and anterior axis polydactyly on the hind limbs and forelimbs of certain embryos, and died 48 h after birth (Luo et al., 1995). A previous study had reported that during evolution, two BMP7 orthologs, *bmp7a* and *bmp7b*, have emerged in some bony fish such as zebrafish (*Danio rerio*) (Shawi and Serluca, 2008). The *bmp7a* and *bmp7b* may have functional differentiation in fish species, loss of BMP7 gene function or acquisition of new functions. Until now, researches on the BMP7 gene in fish is limited, and the dynamic changes during fish development are unclear. In zebrafish, *bmp7a* could form heterodimers with other proteins in the same family and played an important role in the development of dorsal-ventral axis of the embryos (Schmid et al., 2000), while *bmp7b* was expressed in several structures, including the digestive system, fin, optic cup and sensory system (Shawi and Serluca, 2008). Although the gene expression data indicated that BMP7 may play a role in the development of many organs and tissues of vertebrates, mostly previous researches had been focused on the eyes and bones development of mammals. The role of BMP7 in teleosts is not clear and needs to be investigated to improve our understanding of BMP7 functions in vertebrates.

Here, we describe the differential expression of *bmp7a* and *bmp7b* in embryonic, juvenile, and adult zebrafish. In order to explore the roles of *bmp7a and bmp7b* in fish development and growth, we produced *bmp7a* and *bmp7b* mutant zebrafish through using CRISPR/Cas9 gene editing technology. The *bmp7a* mutants died during the gastrulation stage due to defective development of dorsal-ventral axis. The *bmp7b* mutants were found to be viable but showed slow growth, and the color of their body and iris became darker throughout the growth and development process. In addition, defects in the retinal structure of the eyes led to slower feeding responses of the *bmp7b* mutant zebrafish. Comparative transcriptome analysis was conducted to reveal the possible molecular regulation about the impact of *bmp7b* on skin melanin production and eye structural defects. The study helps us understand the functional differentiation of *bmp7a* and *bmp7b* in teleosts and may provide a new perspective for studying the molecular mechanism of fish pigmentation.

## Materials and Methods

All animals experiments were approved by the Institutional Animal Care and Use Ethics Committee of Huazhong Agricultural University (HZAUDO-2016-005, 2016-10-26) and conducted in accordance with the “Guidelines for Experimental Animals” of the Ministry of Science and Technology (Beijing, China).

### Phylogenetic Analysis and Gene Structure Analysis

The sequences of all species (*Homo sapiens*, *Mus musculus*, *Gallus*, *Xenopus tropicalis*, *Danio rerio*, *Latimeria chalumnae*, *Oryzias latipes*, *Oreochromis niloticus*, *Takifugu rubripes*, and *Gasterosteus aculeatus*) for the phylogenetic analysis were obtained from NCBI (https://www.ncbi.nlm.nih.gov). All the sequence IDs are available in Supplementary Table S1. GSDS2.0 (http://gsds.gao-lab.org/index.php) and MEME (https://meme-suite.org/meme/tools/meme) were used to reveal the homology of gene structure among species. The SWISS_MODEL (https://swissmodel.expasy.org/interactive) was used to predict the protein tertiary structures of *bmp7a* and *bmp7b*.

### Generation of Mutant Zebrafish Lines and Maintenance

The transgenic zebrafish parent labeled with green fluorescent protein for osteoblast-specific transcription factor (Osterix GFP) used in this experiment was provided by Professor Xiao Chongde from Chung Yuan Christian University, Taiwan. Fish were kept at 26–28°C under a controlled light cycle (14 h light, 10 h dark) to induce spawning. CRISPR-Cas9 genome editing technology was used to target zebrafish *bmp7a* and *bmp7b*. The sequencing of gRNAs and PCR primers are listed in Supplementary Table S2. The mutations of *bmp7a* and *bmp7b* were predicted to cause a translational frame shift and induced premature transcription termination.

### *In Situ* Hybridization

The zebrafish embryos were fixed with 4% paraformaldehyde for 24 h. Fragments (length between 300 and 600 bp) of *bmp7a* and *bmp7b* were cloned using the primers shown in Supplementary Table S2. The PCR products were purified, and then the whole-mount *in situ* hybridization probes were synthesized in accordance with the manufacturer’s instructions using the DIG RNA Labeling Kit (SP6/T7) (Roche Diagnostics GmbH, Mannheim, Germany, SKU: 11175025910). The steps of whole-mount *in situ* hybridization were performed according to the method described by Thisse and Thisse (2008). Anti-DIG-AP,Fab fragments (Roche, SKU: 11093274910) from Sigma-Aldrich were used as antibodies.

### Histology Analysis

The eye tissues at 30 days post-fertilization (dpf) and skin tissues of wild types and *bmp7b* mutants at 60 dpf (3 samples each) were fixed in 4% paraformaldehyde for 24 h. After that, the samples were dehydrated through a series of graded ethanol solutions (70–100%), embedded in paraffin, and sectioned at 5 μm thickness. These sections were stained with hematoxylin and eosin stain; then analyzed and photographed using an Olympus BX53 Upright microscope (Olympus, Beijing, China).

### Bone Staining

Bone staining with alizarin red was performed as previously described (Nie et al., 2019) for 90 dpf wild types and *bmp7b* mutants (10 samples each). The stained specimens were analyzed and photographed under an Olympus SZX2 Stereo microscope (Olympus, Beijing, China).

### Determination of Skin Melanin Content

To study the change in melanin content, we stripped the skin (0.2 g) of wild type and mutant fish (20 samples each) after anesthetizing, and extracted the melanin referring to a modified method (Yuan et al., 2008). A UV spectrophotometer UV-3100 (Mapada, Shanghai, China) was used to measure the photometric value of the solution containing melanin. Melanin content was expressed as A_290_ nm/g (wet weight).

### Behavioral Observation

Observations of feeding behavior was performed according to previously study (Miller et al., 1993). Fish were starved for 12 h before the experiments. Wild types and *bmp7b* mutants at 90 dpf were separately transferred to an experimental tank (length 30 cm × height 24 cm × width 12 cm filled with 2 L of clean water) and allowed to acclimatize to the environment for at least 1 h. On one side of the tank, 5 ml of brine shrimp (*Artemia salina*) was added at a speed of 15 ml/min to stimulate the fish. A digital video format (Sony, Shanghai, China) was used to record videos of 5 min before and after adding brine shrimp, and EthoVision XT 14 software (Noldus Information Technology, Beijing, China) was used to analyze the behavioral changes (speed, swimming route and reaction time) between the wild-type and mutant fish. The wild type and mutants each had six biological replicates. According to report on zebrafish behavior from Fero et al. (2011), we tested the photosensitivity of the wild-type and mutant fish. Wild types and mutants (all 5 dpf) were separately transferred to an experimental tank (diameter 35 mm, height 10 mm) and allowed to acclimatize to the environment for at least 15 min in the light. Then the light was turned off and the behavior of the fish was recorded. All experiments were performed in EthoVision XT 14 software and there were six samples for wild-type and mutant fish, respectively. During the experiments, the room and water temperatures were maintained at 25 ± 1°C.

### Comparative Transcriptome Analysis

Total RNA was isolated from the eyes and skin of 30 dpf wild types and *bmp7b* mutants with three biological replicates using RNAiso Plus Reagent (TaKaRa, Beijing, China) according to the manufacturer’s protocol. Each sample consisted of tissues from six fish. Subsequently, total RNA was quantified using a 2100 Bioanalyzer system (Agilent Technology, CA, United States) and the samples that met the quality requirements were used for RNA-seq library preparation. After RNA quality and quantity control, 12 transcriptome sequencing libraries were generated using NEBNext® Ultra™ RNA Library Prep Kit for Illumina® (NEB, Beijing, China) following the manufacturer’s recommendations, and index codes were added to attribute sequences to each sample. After cluster generation, the library preparations were sequenced on an Illumina Hiseq platform. After quality control of raw data, paired-end clean reads were aligned to the zebrafish reference genome (ftp://ftp.ensembl.org/pub/release-92/fasta/danio_rerio/) using Hisat2 v2.0.4 (Kim et al., 2015). HTSeq v0.9.1 was subsequently used to count the read numbers mapped to each gene, and then the FPKM of each gene was calculated (Trapnell et al., 2010). Differential expression analysis of two conditions/groups (three biological replicates per condition) was performed using the DESeq R package (1.18.0) (Wang et al., 2009). Genes with a *p*-value < 0.05, adjusted using the Benjamini and Hochberg’s approach for controlling the false discovery rate, were defined as differentially expressed genes (DEGs). KEGG enrichment analysis of DEGs was performed and KOBAS software was used to test the statistical enrichment of DEGs in the KEGG pathways (http://www.genome.jp/kegg/) (Mao et al., 2005).

### Quantitative Real-Time PCR

The NCBI primer tool (https://www.ncbi.nlm.nih.gov/tools/primer-blast/) was used to design primers for the tested genes (*rgs9a*, *rgs9b*, *rcvrn2*, *guca1d*, *grk1b*, *opn1mw4, gc2*, *wnt7ba*, *gna14*, *erbb3b*, *bambia*, *bmp7b*, *β-actin*, *gh1*, *ghra/b*, *igf-1/2a/2b*, *igf1ra/b*, and *igf2r*). We used the PrimeScript RT Reagent Kit with gDNA Eraser (TaKaRa, code No. RR047A) to synthesize the cDNA. The qRT-PCR experiments were performed using TB Green^®^ Premix Ex Taq™ II (Tli RNaseH Plus) (TaKaRa, code No. RR820A) and the QuantStudio™ ^6^ Flex qRT-PCR system (ABI, Foster City, CA, United States). Thermal cycling conditions were as follows: 5 min at 95°C, followed by 40 cycles at 95°C for 20 s, and at 60°C for 25 s. Using the *β-actin* gene of zebrafish as a reference gene, we used the comparative 2^−△△Ct^ method to determine the relative gene expression between the wild types and mutants (Livak and Schmittgen, 2001). IBM SPSS Statistics software (version 22.0) was used to perform a Student’s t-test to compare the differences between the two groups. Statistical significance was set at *p* < 0.05, and statistical high significance at *p* < 0.01. The fold-change values are the average of three biological replicates in each group. The primer sequences are listed in Supplementary Table S2.

## Results

### Phylogenetic Analysis of BMP7 in Vertebrates

**FIGURE 1:**
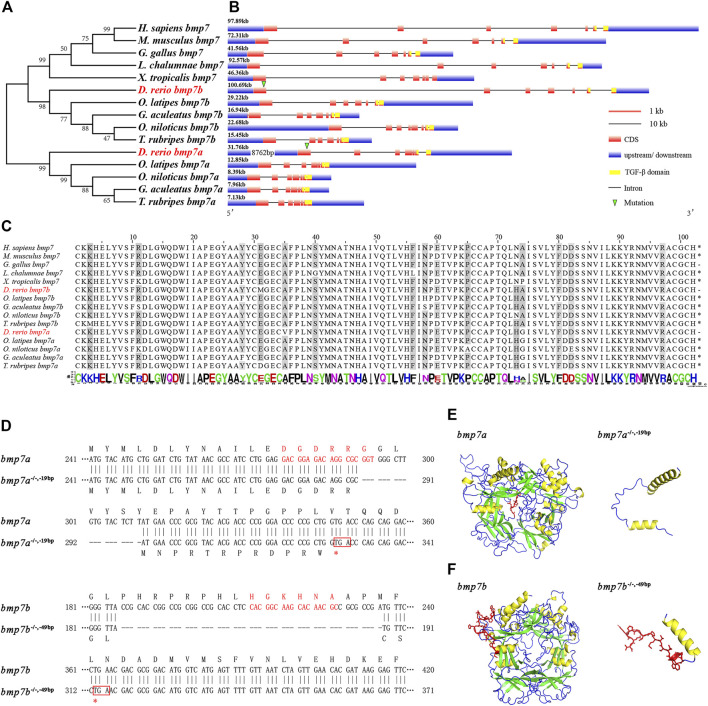
Phylogenetic analysis, conservative sequence, and protein structure prediction of BMP7 genes. **(A)** Phylogenetic analysis of BMP7 genes among different species. **(B)** The structural analysis of BMP7 genes among different species. The green triangle represents the knockout site of *bmp7a* and *bmp7b*. **(C)** Conservation analysis of amino acids in BMP7 genes’ domains among different species. **(D)** Schematic representation of *bmp7a* and *bmp7b* DNA and protein sequences, and the mutant alleles for *bmp7a* and *bmp7b*. CRISPR-Cas9 target sequence is marked with red letters in the wild and mutant DNA sequences. The missing nucleotides are represented by (-) in the mutant, and the red box represents the stop codon. **(E)** The *bmp7a* protein tertiary structure prediction of wild type and mutation with -19 bp deletion. The yellow area represents the α-helix; the green area represents the β-sheet; the red area represents the missing part of the mutation. **(F)** The *bmp7b* protein tertiary structure prediction of wild type and mutation with -49 bp deletion. The yellow area represents the α-helix; the green area represents the β-sheet; the red area represents the missing part of the mutation.

The evolutionary origin and phylogenetic distribution of BMP7 showed that BMP7 orthologs, which evolved from the same ancestral gene family of vertebrates, had duplications in teleosts after the evolution of *L. chalumnae*, named *bmp7a* and *bmp7b*. The *bmp7b* clade was clustered into one group of humans and mammals (Figure 1A). The gene sequence and structure analysis of BMP7 genes showed that the zebrafish *bmp7b* gene is highly homologous to the BMP7 gene in humans and mammals. Although the exon numbers of these genes were the same, zebrafish *bmp7b* (100.69 kb) was closer in length to human (97.87 kb) and mouse (72.31 kb) BMP7 genes, and was significantly larger than zebrafish *bmp7a* (31.76 kb) (Figure 1B). In addition, these species have a common sequence encoded by exon 6 and exon 7, called the TGF-β domain (103aa). The TGF-β domain-encoding sequences were highly conserved across different species (Figure 1C).

### Generation of *bmp7a* and *bmp7b* Mutant Zebrafish

To explore the role of BMP7 gene in fish species and the differences between orthologous genes, we constructed *bmp7a* and *bmp7b* mutant zebrafish using CRISPR-Cas9 technology. One *bmp7a* nonsense allele with a 19 bp deletion in the first exon and two *bmp7b* nonsense alleles with 49 and 25 bp deletions in the first exon were generated. The *bmp7a* and *bmp7b* nonsense alleles all led to premature translation termination (Figure 1D). Additionally, protein structure prediction showed that the TGF-β domains of *bmp7a* and *bmp7b* were destroyed because of the missing sequence and could not form a complete protein structure (Figures 1E,F). Since the phenotypic consistency of different mutant lines of the same gene was confirmed in subsequent experiments, the homozygous mutant lines of *bmp7a* and *bmp7b* were referred to as *bmp7a* ^*-/-*^ and *bmp7b* ^-/-^, respectively, unless otherwise stated.

### *Bmp7a* Deletion Causes Early Embryonic Lethality

**FIGURE 2:**
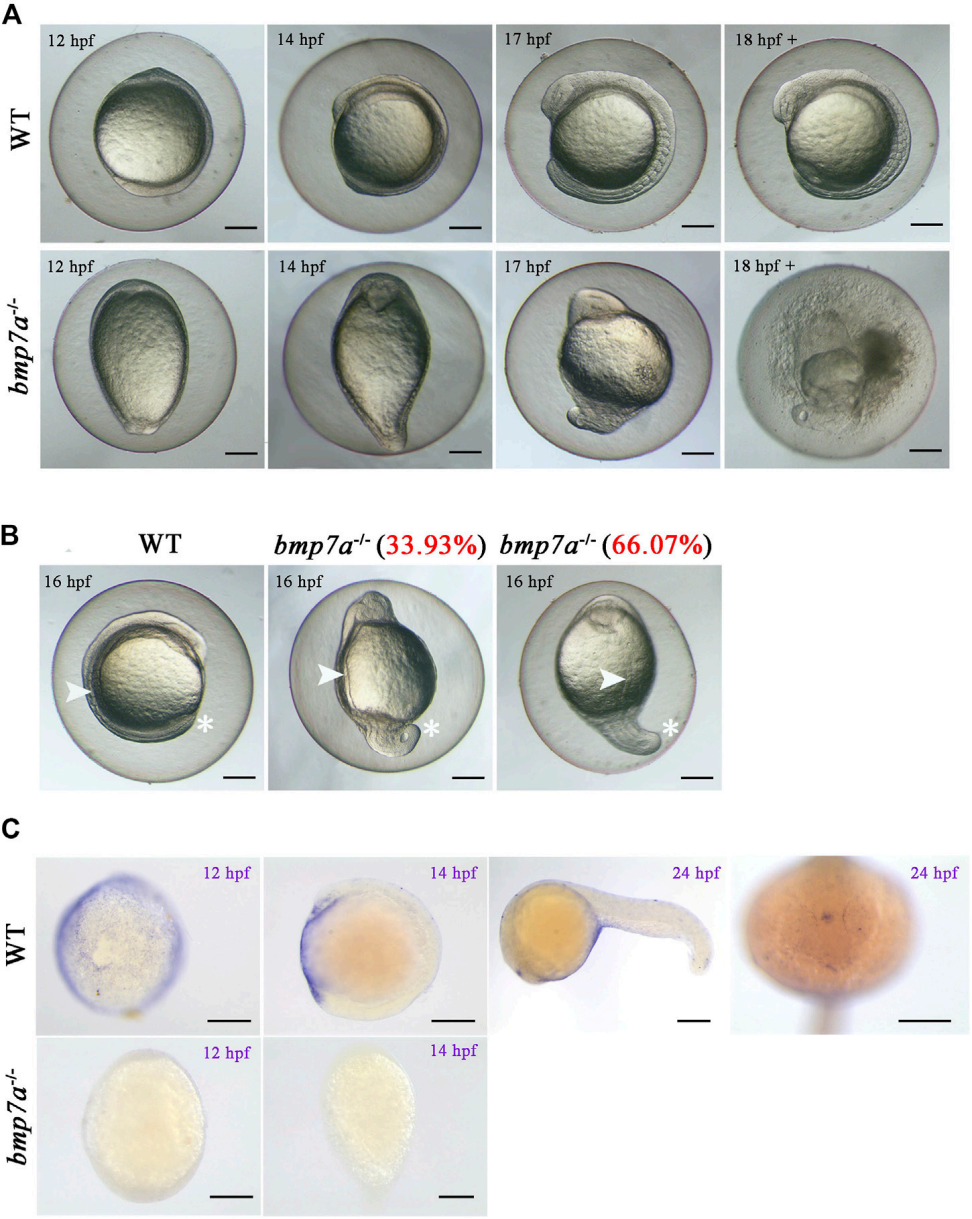
Observation of the homozygous lethal phenotype and gene expression in *bmp7a* embryos. **(A)** Morphological features of the wild-type and *bmp7a* ^-/-^ embryos at 12 hpf, 14 hpf, 17 hpf and after 18 hpf. **(B)** Morphological features of the wild-type and *bmp7a* ^-/-^ embryos at 16 hpf. The proportion of mutant embryos with different morphology in mutant embryos at 16 hpf was shown in red font. **(C)** Whole-mount *in situ* hybridization of the *bmp7a* gene at 12 hpf, 16 hpf wild-type and *bmp*7a^-/-^ embryos, and whole-mount *in situ* hybridization of the *bmp7a* at 24 hpf wild-type embryos. Scale bars, 200 μm.

We did not find any mutants when we tested the 48 hpf embryos from the *bmp7a* heterozygote cross. Therefore, we suspected that *bmp7a* deletion is lethal and would cause early embryonic death in the *bmp7a* ^-/-^ mutants. To confirm this, we detected embryos at different stages of development, and all genotypes containing a homozygous knockout of *bmp7a* were found to be lethal at 12–24 hpf. After all the surviving embryos were tested, no homozygous embryos were found to survive at 48 hpf (Table 1). To elucidate how a homozygous *bmp7a* deletion causes early embryonic death, we examined the morphology of the wild-type and *bmp7a* ^-/-^ embryos. After a large-scale screening of the 501 embryos from three pairs of heterozygous parents, we counted the number of three genotypes. The proportions (*bmp7b* ^+/+^: *bmp7b* ^+/-^: *bmp7b* ^-/-^ = 24.55%: 48.70%: 26.75%) of genotypes met Mendel’s laws of inheritance. Moreover, we simultaneously observed the morphology of these embryos and found that *bmp7a* ^-/-^ embryos had defects in the dorsal-ventral pattern. The *bmp7a* ^-/-^ embryos were morphologically distinguishable as distinctly ovoid-shaped embryos at 11 hpf. In the initial stage of somite formation (13–15 hpf), all somites of the mutants extended to the ventral side, whereas the tail did not develop to the ventral side. At the 10-somite stage (16 hpf), the ventral fusion of somites caused the yolk cells to rupture and eventually led to death (Figure 2A). In addition, the tails of *bmp7a* ^-/-^ mutants showed severe deformities at 16 hpf. Approximately, 34% of the *bmp7a* ^-/-^ mutants developed their tails to the ventral side, 66% to the dorsal side (Figure 2B). Moreover, we investigated the spatiotemporal expression pattern of *bmp7a* using whole-mount *in situ* hybridization. In 12 hpf wild-type embryos, *bmp7a* was expressed on the entire blastoderm, especially on the ventral side. In 14 hpf wild-type embryos, the head and tail ends were significantly expressed, but no expression was observed in the dorsal area. In 24 hpf wild-type embryos, *bmp7a* was significantly expressed in the head and abdomen, but not in the tail. However, in the mutant embryos, the expression of *bmp7a* disappeared at alive time points (Figure 2C).

**TABLE 1:** Genotyping statistics for dead and alive embryos at 12 hpf, 24 hpf and 48 hpf.

Time	12 hpf			24 hpf			48 hpf		
	*bmp7a* ^+/+^	*bmp7a* ^+/-^	*bmp7a* ^-/-^	*bmp7a* ^+/+^	*bmp7a* ^+/-^	*bmp7a* ^-/-^	*bmp7a* ^+/+^	*bmp7a* ^+/-^	*bmp7a* ^-/-^
Dead embryos	27	47	28	4	1	106	0	0	0
Alive embryos	_	_	_	_	_	_	92	196	0
Total	*bmp7a* ^+/+^: *bmp7a* ^+/-^: *bmp7a* ^-/-^ = 123 : 244: 134 = 24.55%: 48.70%: 26.75%								

### *Bmp7b* ^-/-^ Mutants Show Bending of the Haemal Arches and Growth Retardation

**FIGURE 3:**
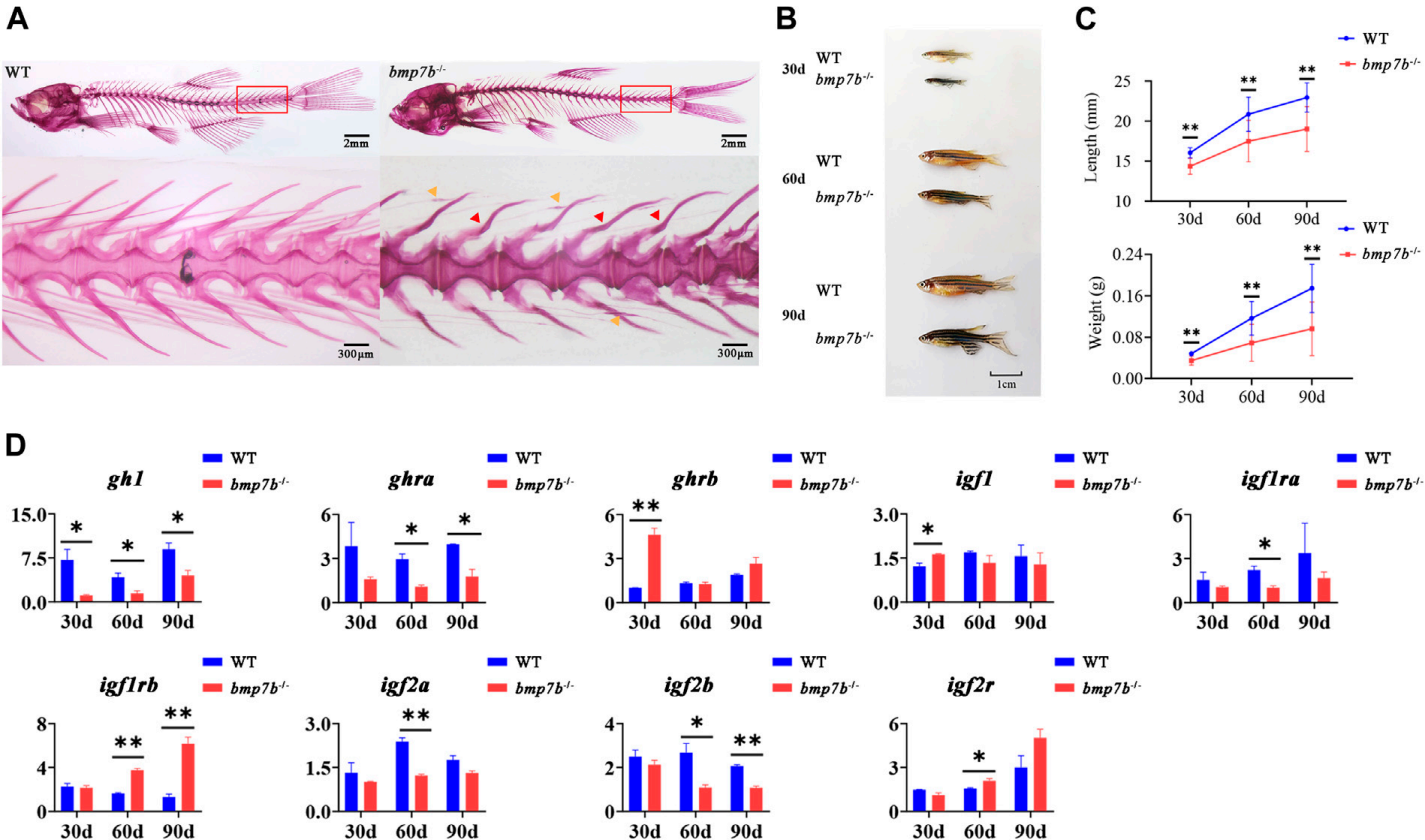
The bone and growth phenotype, and gene expression differences between the wild-type and *bmp7b* ^-/-^ zebrafish. **(A)** Alizarin red staining for mineralized bones at 3 months old fish. The red arrow indicates the severely curved part of the haemal arches, and the yellow arrow indicates the nodular part of the intermuscular bone. **(B)** The external physical characteristics. **(C)** The differences of body length and weight. **(D)** Quantitative expression analysis of growth related genes. Statistically significant differences are marked as *p < 0.05 and **p < 0.01.

We studied the effect of the *bmp7b* gene on bone development by staining adult fish with whole-mount alizarin red. The results showed that approximately 80% of adult *bmp7b* ^-/-^ (*n* = 20) had bend haemal arches and nodules in the intermuscular bones (Figure 3A). Compared to the wild type fish, the external morphology of *bmp7b* ^-/-^ mutants was significantly different. That is, the growth was significantly slower, and these mutants had a darker body surface color (Figure 3B). To compare the effects of *bmp7b* deletion on growth and development, we measured the growth data (body length and weight) of the *bmp7b* ^-/-^ and wild-type zebrafish at three time points (30, 60, and 90 dpf). The results showed that the body length and weight of the *bmp7b* ^-/-^ zebrafish were significantly lower than those of wild types (*p* < 0.05) (Figure 3C).

Fish growth is mainly regulated by the hypothalamus-pituitary-liver growth axis (GH/IGF axis). To explore the causes for the differences in growth between the wild-type and *bmp7b* ^-/-^ zebrafish, we compared the expression levels of genes related to growth (growth hormone 1, *gh1*; growth hormone receptor a/b, *ghra/b*; insulin-like growth factor-1/2a/2b, *igf-1/2a/2b*; insulin-like growth factor 1 receptor a/b; *igf1ra/b*; and insulin like growth factor 2 receptor; *igf2r*) at different points (30, 60, and 90 dpf). The results showed that in the *bmp7b* ^-/-^ mutants, the expression of *gh1* decreased significantly at all three points, and *ghra* and *igf2b* decreased significantly at both 60 and 90 dpf (*p* < 0.05); however, *igf-1* and *ghrb* were increased significantly at 30 dpf, and *igf1rb* was increased at both 60 and 90 dpf (Figure 3D).

### *Bmp7b* ^-/-^ Mutants Exhibit Color Darkening

**FIGURE 4:**
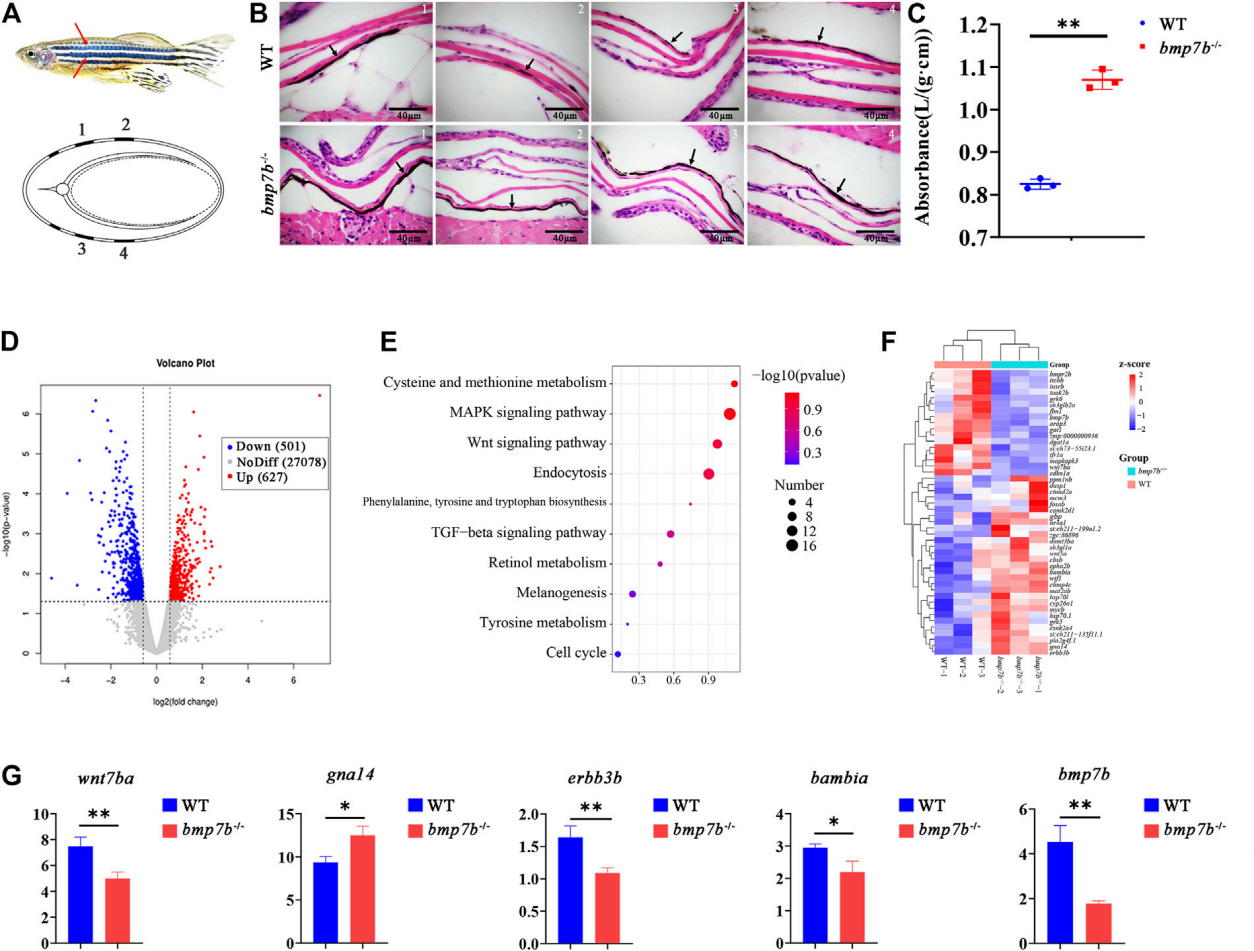
The skin structure, melanin content and gene expression differences between the wild-type and *bmp7b* ^-/-^ zebrafish. **(A)** Schematic diagram. Top: The part of skin melanin tissue section. The red arrow represents the position on the fish body where the tissue section was observed and analyzed. Bottom: the cross-sectional schematic diagram, 1, 2, 3, and 4 correspond to the observation position of the tissue section picture in turn. **(B)** Skin tissue section. The arrow points to the melanin area. **(C)** Determination of the melanin content of the skin. The absorbance/wet weight (A_290_/g) represents the melanin content. **(D)** The volcano map of DEGs. The abscissa represents multiple changes in gene expression in wild-type and *bmp7b* ^-/-^ fish. The ordinate represents the statistical significance of differences in gene expression. **(E)** KEGG enrichment analysis of skin transcriptome. **(F)** Heat map of differential genes enriched in skin transcriptome KEGG. **(G)** qPCR of key genes (*wnt7ba*, *gna14*, *erbb3b*, *bambia* and *bmp7b*). Statistically significant differences are marked as *p < 0.05 and **p < 0.01.

We were intrigued by the darker body color of *bmp7b* ^-/-^ mutants; therefore, we stained the transverse section of the skin at 30 dpf with HE. We compared the structure of the stripes in *bmp7b* ^-/-^ mutants and wild types in four parts (Figure 4A), and found that the skin of the *bmp7b* ^-/-^ mutants had a higher number of melanocytes with a wider distribution (Figure 4B). In addition, the melanin content in the skin of adult *bmp7b* ^-/-^ mutants and wild types were measured. The melanin content in the skin of wild types was 32.987 ± 0.477 A_290_/g, and that in the *bmp7b* ^-/-^ mutants was 42.800 ± 0.904 A_290_/g, indicating a significant difference between the both (*p* < 0.05) (Figure 4C). These results confirmed that the increase in melanin content in the skin of *bmp7b* ^*-/-*^ mutants led to the darkening of the body color.

To further explore the causes for the difference in body color, we compared the transcriptomes of the skin of wild types and *bmp7b* ^-/-^ mutants at 30 dpf using RNA-seq. After quality control of the original data, reference genome mapping, and gene expression level quantification, a total of 1128 DEGs between the two groups, including 627 upregulated genes and 501 downregulated genes in mutants, were identified based on an adjusted *p*-value < 0.05 (Figure 4D, Supplementary Table S3). Subsequently, KEGG pathway analysis revealed important signalling pathways that were significantly enriched in DEGs, such as the mitogen-activated protein kinase (MAPK) (KO: dre 04010), Wnt (KO: dre 04310), melanogenesis (KO: dre 04916), and tyrosine metabolism (KO: dre 00350) signaling pathways, associated with melanin production (Figure 4E, Supplementary Table S4). The upregulation of *gna14* and downregulation *of wnt7ba, erbb3b,* and *bambia* might promote an increase in melanin production in the skin (Figure 4F). The expression of candidate genes was verified by qPCR (Figure 4G), and the results were consistent with the transcriptome data.

### *Bmp7b* ^-/-^ Mutants Have Abnormal Eye Development

**FIGURE 5:**
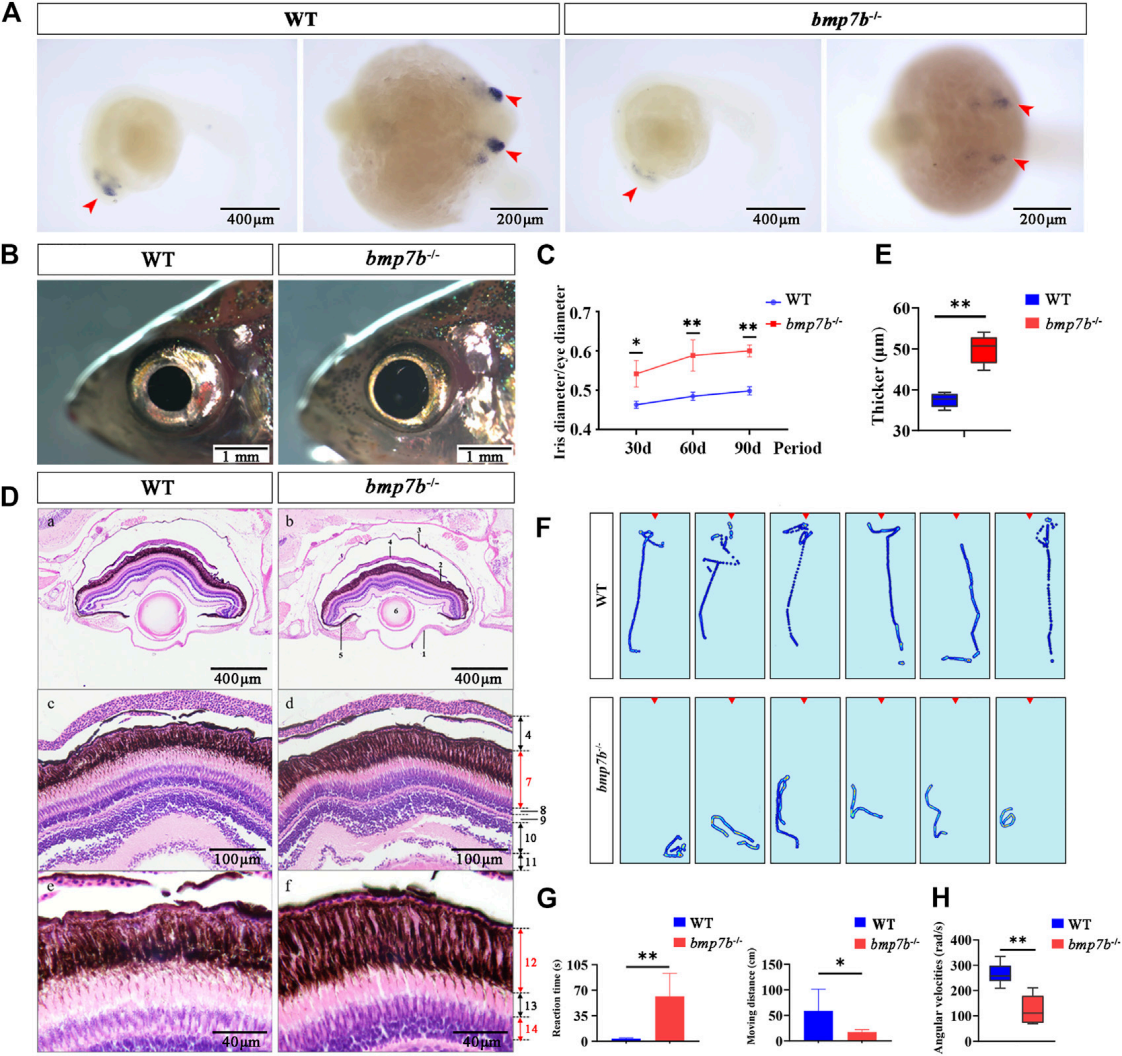
The eye structure, feeding behavior and gene expression differences between the wild-type and *bmp7b* ^-/-^ zebrafish. **(A)** Whole-mount *in situ* hybridization of the *bmp7b* at 24 hpf. The blue area represents the zebrafish eyes. **(B)** The external physical characteristics of eyes. **(C)** Comparative analysis of the proportion of iris diameter in eye diameter in different points (30, 60, and 90 dpf). **(D)** HE staining results of eye tissue sections. 1, cornea; 2, retina; 3, sclera; 4, choroid; 5, iris; 6, lens; 7, pigment cell epithelium and photosensitive layer; 8, outer granular layer; 9, outer reticulum layer; 10, inner granule Layer; 11, inner reticulum layer; 12, pigment cell layer; 13, rod cell layer; 14, cone cell layer. **(E)** Thickness measurement of melanin layer in different regions of eye tissue slices (*n* = 6). **(F)** Heat map showing trajectories of adults within 5 s after stimulation (adding brine shrimp) (six repetitions). The red triangle area represents the location of adding brine shrimp. **(G)** Reaction time and movement distance of adult zebrafish within 5 s after adding brine shrimp. **(H)** Angular velocities of juvenile zebrafish within 5 s after the light intensity was reduced (from light to dark). Statistically significant differences are marked as *p < 0.05 and **p < 0.01.

The results of whole-mount *in situ* hybridization of wild-type embryos showed that *bmp7b* was significantly expressed in the eyes in 24 hpf embryos, confirming that *bmp7b* may have a role in eye development in fish. Additionally, we also observed that the expression of *bmp7b* in the mutants was significantly reduced (Figure 5A). The eyes of *bmp7b* ^-/-^ mutants showed a larger iris (Figure 5B), and the ratio of the iris diameter to the eye diameter also increased significantly (*p* < 0.05) and gradually increased with age (Figure 5C).

Furthermore, we observed the histological characteristics of the eyes in wild-type and *bmp7b* ^-/-^ zebrafish at 60 dpf by longitudinal tissue sections and found an increase in the thickness of the pigment cell layer of the *bmp7b* ^-/-^ mutants (Figures 5D,E). Therefore, we speculated that the changes in the retinal structure caused by *bmp7b* deletion may impact the vision of *bmp7b* ^-/-^ mutants. To verify this hypothesis, we analyzed the feeding behavior of *bmp7b* ^-/-^ mutants by observing their reaction after the addition of food (brine shrimp). The response of the wild types to food was instant while that of the *bmp7b* ^-/-^ mutants was more sluggish (Figure 5F), consistent with their trajectory behavior. After adding brine shrimp, the wild types quickly found it and swam towards it, while the *bmp7b* ^-/-^ mutants took a longer time to discover it. Additionally, we also analyzed the behavior 5s after adding brine shrimp and the sensitivity to food and the moving distance of *bmp7b* ^-/-^ mutants was significantly lower than that of the wild types (*p* < 0.05) (Figure 5G). In order to further verify whether the eye development of the mutant was affected, we examined the angular velocities of wild type and *bmp7b* ^-/-^ mutants within 5 s after the light intensity was reduced (from light to dark). The results showed that the angular velocity of the mutant was significantly reduced (*p* < 0.05) (Figure 5H).

**FIGURE 6:**
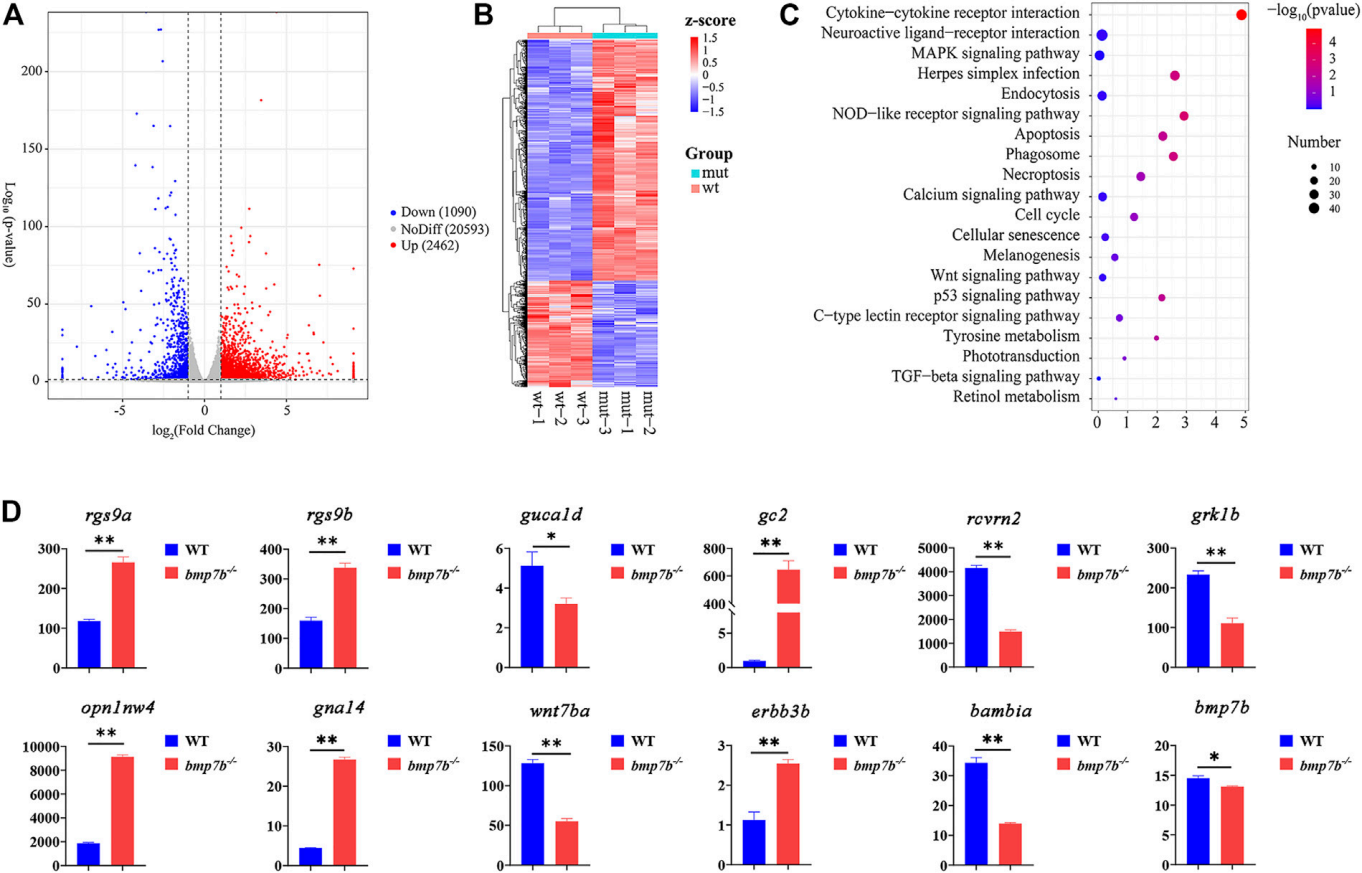
The transcriptome and gene qRT-PCR analysis of eye tissues between the wild-type and *bmp7b* ^-/-^ zebrafish at 30 days. **(A)** DEG’s volcano map. **(B)** DEG’s Heat map. **(C)** KEGG enrichment analysis of the eye transcriptome. **(D)** QPCR of key genes (*rgs9a*, *rgs9b*, *rcvrn2*, *guca1d*, *grk1b*, *opn1mw4, gc2,* and *bmp7b*). Statistically significant differences from the wildtypes and *bmp7b* ^-/-^ mutants are marked as *p < 0.05 and **p < 0.01.

The transcriptomes of the eye tissues between the wild-type and *bmp7b* ^-/-^ zebrafish at 30 dpf were compared using RNA-seq. Transcriptomic analysis indicated significant differences between the wild-type and *bmp7b* ^-/-^ zebrafish (Figure 6A). Over 3,552 DEGs were detected in the eyes of *bmp7b* ^-/-^ fish compared to wild types, with 1,090 DEGs were upregulated and 2,462 DEGs were downregulated (Figure 6B, Supplementary Table S5). KEGG analysis showed that 20 enriched pathways for the DEGs between wild-type and *bmp7b* ^-/-^ mutants were clustered. These included six pathways related to diseases or immune responses (Supplementary Table S6), including apoptosis (KO: dre00010), necroptosis (KO: dre04217), phagosome (KO: dre04145), cell cycle (KO: dre04110), cellular senescence (KO: dre04218), and herpes simplex infection (KO: dre05168), suggesting that inflammatory and eye or retina-related diseases appeared during the development of the eye. In addition, changes in the structure of the eye and disease pathways may lead to the changes in phototransduction (KO: dre04744), which has been associated with dramatic morphological, structural, and functional changes in eye or retinal development. This resulted in a decrease in the vision of the mutants, which was also consistent with the behavioral results. Wnt (KO: dre04310), TGF-β (KO: dre04350), tyrosine metabolism (KO: dre00350), and MAPK signaling pathways (KO: dre04010) were all involved in the production and development of melanin (Figure 6C). *Rgs9a*, *rgs9b*, *rcvrn2*, *guca1d*, *grk1b*, *opn1mw4,* and *gc2* genes might be associated with the light-sensing ability of the eye, while *wnt7ba*, *gna14*, *erbb3b*, and *bambia* genes might promote an increase in melanin in the iris of the eyes. The expression of candidate genes was verified by qPCR (Figure 6D), and the results were consistent with the transcriptome data.

## Discussion

Compared to mammals, birds and amphibians, which have only one BMP7 gene, fish-specific genome duplication (FSGD) led to the formation of BMP7 homologs*, bmp7a* and *bmp7b,* in zebrafish and other Actinopterygii. *L. chalumnae*, which belongs to Sarcopterygii, only has one BMP7 gene. Therefore, it was speculated that BMP7 homologs might have appeared after the differentiation of the Actinopterygii and the Sarcopterygii. Previous genome replication studies have shown that gene homologs might lose, silence, or gain new functions (Lynch and Force, 2000; Glasauer and Neuhauss, 2014). For example, although the *dmrt2a* and *dmrt2b* domains are highly conserved in zebrafish, the functions of these two genes are not consistent. *Dmrt2a* is required for symmetric somite formation, whereas *dmrt2b* regulates somite differentiation, impacting slow muscle development in zebrafish (Johnsen and Andersen, 2012). Similar to *dmrt2*, the sequence homology analysis of *bmp7a* and *bmp7b* proteins revealed that the protein similarity reached 70%, and the structural domain was highly conserved. However, the significantly reduced gene scale and changed protein coding of the *bmp7a* gene (*bmp7a*, 3384 bp; *bmp7b*, 1694 bp) indicate that the gene is likely to evolve into new functions. Our results showed that *bmp7a* was involved in the development of the dorsal-ventral pattern; while *bmp7b* was involved in the development of eyes and bones, which also mediates the production of skin melanin, indicating that *bmp7b* has a new function in fish species.

The zebrafish snailhouse mutants displayed the strongest dorsalization in the dorso-ventral patterning during gastrulation, which was verified by morpholino knockdown study that *bmp7a* mutation caused the dorsalized phenotype (Lele et al., 2001). The precisely coordinated induction and morphogenesis processes of gastrulation establish the basis of vertebrate bodies (Keller et al., 2000). BMP2, BMP4, and BMP7 have crucial roles in the formation of the dorsal-ventral pattern of the early embryonic mesoderm. Previous studies on *Xenopus laevis* have shown that overexpression of BMP7 led to ventralization of early embryos and loss of dorsal tissues (Graff et al., 1996). The *bmp7a* ^-/-^ mutants that we constructed died during the segmentation period due to severe dorsal-ventral axis defects in the embryos. The mutants continued to develop in an ovoid shape after gastrulation, then the dorsal somites and tails of *bmp7a* ^-/-^ mutants could not develop normally. At the same time, *bmp7a* was abundantly expressed in the abdomen of early embryos, indicating that *bmp7a* plays a key role in the development of the dorsal-ventral pattern of early embryos. Interestingly, BMP signaling is involved in the growth and pattern formation of the brain (Miyake et al., 2017). We found *that bmp7a* was not only expressed in the abdomen but also in the brain area of wild-type embryos at 24 hpf. Our results suggest that *bmp7a* is involved in the formation of the dorsal-ventral pattern of the early embryonic mesoderm, as well as the development of the brain during the embryonic period.

BMP7 is also a growth factor and a secretory signaling molecule. Previous studies have shown that BMP7 is a possible candidate gene for bone and body growth characteristics of chicken (*Gallus gallus*) and large yellow croaker (*Larimichthys crocea*) (Wang et al., 2018; Zhou et al., 2019). Compared with the wild types, *bmp7b* ^*-/-*^ mutants showed a significant decrease in body length and weight during growth (*p* < 0.05). In contrast to other vertebrates, most fish continue to grow during their life cycle (Johnston et al., 2011). In fish, growth hormones (*gh*) are synthesized by the pituitary and released into the blood, then act on certain organs by binding to the growth hormone receptor (*ghr*) on the target cell membrane to regulate growth and development (Kittilson et al., 2011; McMenamin et al., 2013; Hu et al., 2019). Our data showed that *bmp7b* knockout in zebrafish resulted in a significant downregulation of *gh1*, and its receptor *ghra* was also significantly downregulated. In addition, the main target tissue of insulin-like growth factors, *igf1* and *igf2*, is the skeletal muscle in mammals (Duan et al., 2010), and they exert their biological effects by binding to *igf1r* (Glass, 2005; Wu and Zhu, 2011). The significantly reduced expression of *igf2a/2b* at 60 and 90 dpf (*p* < 0.05) might lead to a slower growth of muscle cells in mutants. Correspondingly, the increased expression of *igf1rb* was due to a negative feedback regulation in response to the downregulation of *igf2a/2b* expression, but it still could not prevent the negative effects of *igf2a/2b* expression decline on muscle growth. In summary, *bmp7b* deletion affected the expression of GH/IGF axis genes, thereby inhibiting the growth of *bmp7b* ^-/-^ mutants.

The importance of the BMP7 signaling pathway in vertebrate retinal development has been repeatedly demonstrated through gene inactivation and functional gain experiments in mice and birds (Hogan, 1996; Zhang et al., 2013). During zebrafish embryonic development, *bmp7b* is first expressed in the eye (Shawi and Serluca, 2008), and it can significantly increase the number of outer nodal protrusions formed by rod-shaped cells and cone cells (Sehgal et al., 2006). We found that pigment layers in the eye tissue sections of *bmp7b* ^-/-^ mutants at 60 dpf was thicker than that of the wild types. Therefore, we hypothesized that the vision of *bmp7b* ^-/-^ mutants was affected. Both adult and juvenile zebrafish depend on their vision for feeding (McElligott and O’Malley, 2005; Howe et al., 2018). Therefore, we observed the feeding behavior of zebrafish and found that the reaction time of *bmp7b* ^-/-^ mutants to food stimulation was much longer than that of the wild type, thereby confirming that *bmp7b* deletion reduced the ability of mutant zebrafish to observe food and the visual sensitivity. To further explore whether the eye development process in mutants was affected, we examined the angular velocities of wild types and *bmp7b* ^-/-^ mutants within 5 s after the light intensity was reduced (from light to dark) (Fero et al., 2011). The angular velocity of the *bmp7b* ^-/-^ mutants was significantly reduced (*p* < 0.05), indicating that the response of the mutants to light intensity was less sensitive than that of the wild types. In addition, reduced swimming speed of mutants also might be due to skeletal defects. Taking together, *bmp7b* ^-/-^ mutants had skeletal defects and visual dysfunction, which led to a reduction in feeding ability.

Moreover, we also found that *bmp7b* deletion resulted in an increase in melanin content in the zebrafish skin and eyes. In human hair and skin, BMP7 protein expression was found to change in a hair cycle-dependent manner, which suggests that BMP7 may be involved in the regulation of skin development and function (Adly et al., 2006). Although BMP7 is not a traditional pigment gene, it directly or indirectly affects some pigmentation pathways. It can additionally inhibit normal melanocyte growth, tumor growth, and metastasis of human uveal melanoma (Notting et al., 2007; Hsu et al., 2008; Na et al., 2009), but its dual role in cancer indicates that high BMP7 expression promotes the development of melanoma (Hsu et al., 2005; Rothhammer et al., 2005). BMP7 can affect the production of pheomelanin by interacting with melanocortin during the differentiation and thermogenesis of brown adipocytes (Baxter et al., 2009; Richard and Picard, 2011). In addition, the upregulation of BMP7 is related to hyperpigmentation of the visceral peritoneum and combs (Luo et al., 2013; Dong et al., 2019). Our results indicated that *bmp7b* inhibited the production of zebrafish melanin, while *bmp7b* knockout suppressed this inhibitory effect and caused an increase in melanin production and darkening of zebrafish body color.

The signaling pathways associated with eye defects of *bmp7b* ^-/-^ mutants were revealed through a comprehensive transcriptomic analysis, and the changes in the expression of many key genes involved in phototransduction. Phototransduction is the process by which light is converted into electrical signals *via* photoreceptors (rods and cones) in the retina, which mediates vision in vertebrates (Fu and Yau, 2007). In this process, the reduction in *rcvrn2* expression accelerates cone photoresponse recovery (Zang et al., 2015). The concentration of *rgs9* is a potentially important determinant of faster response kinetics and lower sensitivity of cones, as compared with rods (Cowan et al., 1998). In *bmp7b* ^-/-^ mutants, the expression changes of *rcvrn2*, *rgs9a,* and *rgs9b* genes affected the sensitivity of light response recovery. The decreased expression of *guca1d* (*gcap4*), which is uniquely present in cone photoreceptors, indicated that its reception of light was hindered in *bmp7b* ^-/-^ mutants (Imanishi et al., 2014), whereas the increased expression of *opn1mw4* in the green cones leads to an inaccurate detection of red-shifted down-welling light in *bmp7b* ^-/-^ mutants (Ogawa and Corbo, 2021). In addition, it is possible that the decreased expression of *grk1b* had an impact on the formation of functional cone mosaics. Therefore, the spatial resolution of zebrafish is affected (Chrispell et al., 2018). The changes in genes related to retinol metabolism (pathway ID: dre00830) also play a pivotal role in phototransduction (Parker and Crouch, 2010; Li et al., 2018). In short, the function of cones and rods was based on the phototransduction, and the deletion of *bmp7b* led to the expression changes of genes related to phototransduction. Therefore, it was suggested that the sensitivity of *bmp7b* ^-/-^ mutants to light was decreased, which in turn affected feeding behavior.

Similar to the eye transcriptome, their differential genes in skin tissues were jointly enriched in signaling pathways, such as the MAPK, Wnt, melanogenesis, and TGF-β signaling pathways. In melanogenesis, *gna14* and *wnt7ba* genes showed the same trend of changes in the skin and eyes, and these changes might be key factors leading to an increase in melanin. The Wnt signaling pathway plays a vital role *via* the microphthalmia-associated transcription factor (*Mitf*) in the development of melanophores (Jin et al., 2001). *Wnt7b* is a direct target of canonical BMP signaling in hair follicle stem cells, and *wnt7b* expression reaches a peak during the development of black and white striped feather hair follicles in the neck of eight-week-old chicken (Kandyba and Kobielak, 2014; Wang et al., 2019). In *Ctenophorus decresii*, *wnt7b* showed stronger expression in skin with a high melanin concentration (Mclean et al., 2017). In addition, *wnt7b* is highly expressed in all benign nevi and some large cell melanomas (Pham et al., 2003). *Gna14* is another important gene involved in melanin production; the cell pellets of newborn human melanocytes with *gna14* mutations were lighter in color than those of wild types (Lim et al., 2016). Moreover, *gna14* is one of the candidate genes that caused differences in coat color between white rex rabbits and purple-blue rex rabbits (Qin, 2014). *Gna14* can also be coupled to melanin and participates in melanopsin phototransduction *in vivo* (Hughes et al., 2015). Melanin production was also affected by changes in gene expression in other pathways; signals mediated by the tyrosine kinase *erbb3b* establish the latent precursors metamorphic melanophores, contributing to melanophore development (Budi et al., 2008). *Bambi* is regulated by TGF-β signaling and is overexpressed in melanoma skin compared to normal skin (Hagedorn et al., 2016). Melanin production involves several signalling pathways and the regulation of transcription factors. In this study, we confirmed that *bmp7b* is a key inhibitor of melanogenesis that exhibits its effects by regulating the expression of the above mentioned genes.

In summary, we constructed two stable mutant strains, *bmp7a* and *bmp7b*, and revealed their functional differentiation. *Bmp7a* plays a vital role in the development of the dorsal-ventral pattern of the embryo, while *bmp7b* impacts eye development and melanin production. The first report about the inhibitory effect of *bmp7b* on melanogenesis may provide useful information for the future research on human melanoma-related diseases.

## Data Availability

The datasets presented in this study can be found in online repositories. The names of the repository/repositories and accession number(s) can be found below: NCBI PRJNA788440.
